# Endemic HBV among hospital in-patients in Bangladesh, including evidence of occult infection

**DOI:** 10.1099/jgv.0.001628

**Published:** 2021-07-30

**Authors:** Fazle Rabbi Chowdhury, Anna L. McNaughton, Mohammad Robed Amin, Lovely Barai, Mili Rani Saha, Tanjila Rahman, Bikash Chandra Das, M. Rokibul Hasan, K. M. Shahidul Islam, M. A. Faiz, Mamun Al-Mahtab, Jolynne Mokaya, Barbara Kronsteiner, Katie Jeffery, Monique I. Andersson, Mariateresa de Cesare, M. Azim Ansari, Susanna Dunachie, Philippa C. Matthews

**Affiliations:** ^1^​ Department of Internal Medicine, Bangabandhu Sheikh Mujib Medical University, Dhaka 1200, Bangladesh; ^2^​ Nuffield Department of Medicine, Peter Medawar Building for Pathogen Research, South Parks Rd, Oxford OX1 3SY, UK; ^3^​ Mahidol-Oxford Tropical Medicine Research Unit (MORU), Bangkok 10400, Thailand; ^4^​ Department of Medicine, Dhaka Medical College, Dhaka 1200, Bangladesh; ^5^​ Department of Microbiology, BIRDEM General Hospital, Dhaka 1200, Bangladesh; ^6^​ Surveillance and Immunization Unit, World Health Organization Office, Dhaka 1200, Bangladesh; ^7^​ Dev Care Foundation, Dhaka 1200, Bangladesh; ^8^​ Department of Hepatology, Bangabandhu Sheikh Mujib Medical University, Dhaka 1200, Bangladesh; ^9^​ Centre for Tropical Medicine and Global Health, Peter Medawar Building for Pathogen Research, South Parks Road, Oxford, OX1 3SY, UK; ^10^​ Department of Microbiology and Infectious Diseases, Oxford University Hospitals NHS Foundation Trust, John Radcliffe Hospital, Headley Way, Headington, Oxford OX1 3SY, UK; ^11^​ Wellcome Centre for Human Genetics, Roosevelt Drive, Headington, Oxford, OX3 7BN, UK; ^12^​ NIHR Biomedical Research Centre, John Radcliffe Hospital, Headley Way, Headington, Oxford OX1 3SY, UK

**Keywords:** Bangladesh, epidemiology, hepatitis B virus, occult, screening, sequencing

## Abstract

Bangladesh is one of the top-ten most heavily burdened countries for viral hepatitis, with hepatitis B (HBV) infections responsible for the majority of cases. Recombinant and occult HBV infections (OBI) have been reported previously in the region. We investigated an adult fever cohort (*n*=201) recruited in Dhaka, to determine the prevalence of HBV and OBI. A target-enrichment deep sequencing pipeline was applied to samples with HBV DNA >3.0 log_10_ IU ml^−1^. HBV infection was present in 16/201 (8 %), among whom 3/16 (19 %) were defined as OBI (HBsAg-negative but detectable HBV DNA). Whole genome deep sequences (WGS) were obtained for four cases, identifying genotypes A, C and D. One OBI case had sufficient DNA for sequencing, revealing multiple polymorphisms in the surface gene that may contribute to the occult phenotype. We identified mutations associated with nucleos(t)ide analogue resistance in 3/4 samples sequenced, although the clinical significance in this cohort is unknown. The high prevalence of HBV in this setting illustrates the importance of opportunistic clinical screening and DNA testing of transfusion products to minimise OBI transmission. WGS can inform understanding of diverse disease phenotypes, supporting progress towards international targets for HBV elimination.

## Introduction

Estimates suggest that approximately a third of the world’s population has been exposed to hepatitis B virus (HBV), with chronic HBV infection (CHB) affecting more than 260 million individuals worldwide, leading to 800 000 deaths annually [1]. Ambitious elimination targets have been established, linked to UN Sustainable Development Goals [2]. Bangladesh has an intermediate CHB prevalence, estimated at 2–6 % [3, 4], although epidemiology varies between regions and according to sociodemographic factors [5]. In combination with the large population, this HBV prevalence puts Bangladesh in the top-ten highest-burdened countries for viral hepatitis worldwide [6], with the perinatal incidence of new infections among the highest in South Asia [7].

HBV prevalence estimates are typically based on HBsAg screening. However, this does not account for occult HBV infection (OBI), in which individuals are HBV DNA positive, but HBsAg negative (usually in combination with a positive anti-HBc antibody) [8]. OBI can arise in several contexts. Typically, when HBsAg is undetectable, corresponding viral loads (VL) are low (<200 IU ml^−1^), reflecting minimal production of HBsAg, or impaired egress from hepatocytes. A number of mutations have been linked to this low-HBsAg phenotype [8–10]. Occult phenotypes may also be driven by anti-HBs seroconversion [11, 12]. Mutations in HBsAg affecting antibody binding in diagnostic assays have also been described, meaning HBsAg is expressed but not detected (‘false-occult’ infections) [8]. This is pertinent for assays that rely on detection of the ‘a’ determinant of the small HBsAg protein [13]; substitutions, insertions and deletions in this region can result in OBI [14].

OBI has been described in Bangladesh in a range of different contexts [15, 16], with case reports of OBI transmission documented [17, 18]. The majority of clinical and epidemiological studies overlook OBI, as HBV DNA screening is expensive. However, recommendations for screening blood products increasingly include HBV DNA testing in order to avoid OBI transmission [19]. Recognising the high burden of HBV infection in Bangladesh, and limited data about OBI, we set out to investigate the prevalence and molecular characteristics of HBV and OBI in a hospital cohort.

## Methods

### Study settings and clinical cohort

We screened a prospective observational fever cohort in Bangladesh to opportunistically study characteristics of HBV infection and OBI. This hospital cohort was recruited to examine causes of febrile illness, and it was anticipated that HBV infection would not be relevant to the diagnosis for which patients had presented. Serum samples were collected from adults (≥18 years of age) recruited between June-October 2017 at two sites:

1. Dhaka Medical College Hospital (DMCH) is a public hospital, and the largest tertiary care hospital in Bangladesh with 2300 beds. Patients are admitted to a full range of specialties, and mostly receive free care.
2. The Bangladesh Institute of Research and Rehabilitation in Diabetes, Endocrine and Metabolic Disorders (BIRDEM), is a non-government not for profit specialist hospital in Dhaka with 750 beds. Baseline investigations are performed at a government-subsidised rate, but patients typically pay for specialist investigations.

Both hospitals receive patients from across the country. Patients from Internal Medicine, Critical Care Medicine, Surgery or Orthopaedics were eligible if presenting with a history of fever >38 °C for >48 h. As routine infant HBV vaccination was not rolled out in Bangladesh until 2003, few of this cohort are likely to have received childhood immunisation against HBV.

### Blood sampling and baseline screening for HBV infection

Serum samples were collected and screened for HBV infection and HBV exposure at local accredited testing facilities (Virology department of Bangabandhu Sheikh Mujib Medical University) in Dhaka, using HBsAg (LIAISON XL Murex HBsAg Quant, Italy) and total antibody to HBV core (anti-HBc) (LIAISON anti-HBc, Italy) respectively. All HBsAg-positive and anti-HBc-positive patients were screened for HBV DNA at the clinical diagnostic laboratory at Oxford University Hospitals NHS Foundation Trust (OUH), UK (COBAS AmpliPrep/COBAS TaqMan, Roche, Welwyn Garden City, UK [20]) with an automated platform to detect and quantify HBV DNA. HBV DNA positive samples also underwent HBeAg testing at OUH, when sufficient sample was available (Abbott Architect i2000SR).

### Clinical follow-up

Patients who tested HBV positive were informed about the result and referred to Hepatology services for clinical assessment, management and follow-up.

### DNA extraction and sequencing

Serum volumes ≤0.5 ml were made available for the study. Samples with HBV DNA VL ≥3.0 log_10_ IU ml^−1^ underwent a target-enrichment approach for HBV whole genome sequencing (WGS) on an Illumina Mi-Seq platform in Oxford, UK. This threshold is determined by the sensitivity of current deep sequencing approaches for WGS [21, 22]. DNA was extracted from serum using the NucliSENS magnetic extraction system (bioMérieux). A completion-ligation reaction was performed to convert the partially dsDNA genome into a fully dsDNA molecule [23], after which DNA was purified using Agencourt RNAClean XP magnetic beads (Beckman Coulter).

Sequencing libraries were generated using the NEBNext Ultra II DNA Library Prep Kit (New England Biolabs) and libraries were assessed using TapeStation system (Agilent) and Qubit dsDNA HS Assay (Thermo Fisher Scientific). An adapted target-enrichment workflow was applied to the SeqCap EZ (Roche) protocol, using custom-designed HBV probes ordered from IDT (xGen Lockdown Probes). Samples were sequenced on an Illumina Mi-Seq using a v3 300 bp paired end kit.

### Analysis of sequence data

Deep sequencing read pairs were de-multiplexed using QUASR v7.01 and adapter sequences trimmed with CutAdapt v1.7.1 [24]. Short reads (< 50 bp length) and reads mapping to the human genome reference sequence (identified using Bowtie v2.2.4 [25] were discarded. Remaining reads were mapped to HBV reference sequences representing genotypes A-I using BWA mem v0.7.10 [26], to select the most appropriate reference sequence and to identify HBV reads. Reads were then mapped against the reference sequence using BWA mem and consensus sequences were derived. Simmonics Sequence Editor [27] was used for alignment and sequence examination, using the genotype A sequence X02763 as a numbering reference. Maximum-likelihood phylogenetic analysis of consensus sequences was performed with mega7 [28] with 1000 bootstrap replicates used. Trees were visualised in Figtree [29]. Sequences were analysed with reference sequences [30] and 61 published full length HBV genome sequences from Bangladesh identified in Genbank.

We looked for evidence of dual infections in the mapping of reads to HBV genotypes A-I and consensus sequences were further checked for evidence of recombination by bootscan analysis using RDP4 [31].

### Identification of potential resistance associated mutations (RAMs) and vaccine escape mutations (VEMs)

We referred to previously published lists of HBV RAMs for lamivudine (3TC) [32] and tenofovir disoproxil fumarate (TDF) [33]. VEMs are most commonly reported within the HBV surface antigen (HBsAg) ‘a’ determinant (residues 124–147), which is the major target of neutralising antibodies. We therefore focused scrutiny on this region, searching for evidence of previously catalogued polymorphisms Q129H/R, M133L, F/Y134I/L/T, K141E, P142S and G145R/A [34]. We also added A128V, as this has been independently reported as a VEM in Bangladesh [35, 36].

### Statistics

Data were analysed using GraphPad Prism version 7.0 (San Diego, CA, USA). Two-sided *P* values were calculated using the Chi-square and Fisher’s exact test for dichotomous and ordinal variables. Continuous variables were compared using one-way ANOVA and the Mann-Whitney *U*-test. Demographic factors and clinical characteristics were summarized with counts (%) for categorical variables and median (interquartile range [IQR]) and mean (±standard deviation) for continuous variables.

## Results

### HBV epidemiology

The cohort recruited 201 adults, with a median age of 35 years (IQR 28–55 years), of whom 115/201 (57 %) were male. Exposure to HBV was common, with 72/201 (35.8 %) testing anti-HBc-positive (Table S1). Active HBV infection was confirmed in 16/201 individuals (7.9 %), comprising 10/16 who were positive for both HBsAg and HBV DNA (62.5 %), 3/16 positive for HBsAg with undetectable HBV DNA (18.8 %), and 3/16 with OBI based on testing positive for HBV DNA with negative HBsAg (18.8 %) (Fig. S1, available in the online version of this article). A clinical diagnosis was available for all patients; leading causes of admission were enteric fever, urinary tract infections and septicaemia (accounting for 60 % of all admissions). There was no difference in reasons for admission between HBV-positive and negative individuals, and liver infection/disease was not recorded as a diagnosis for any patient (see Metadata Table, https://doi.org/10.6084/m9.figshare.11973930).

**Fig. 1.**
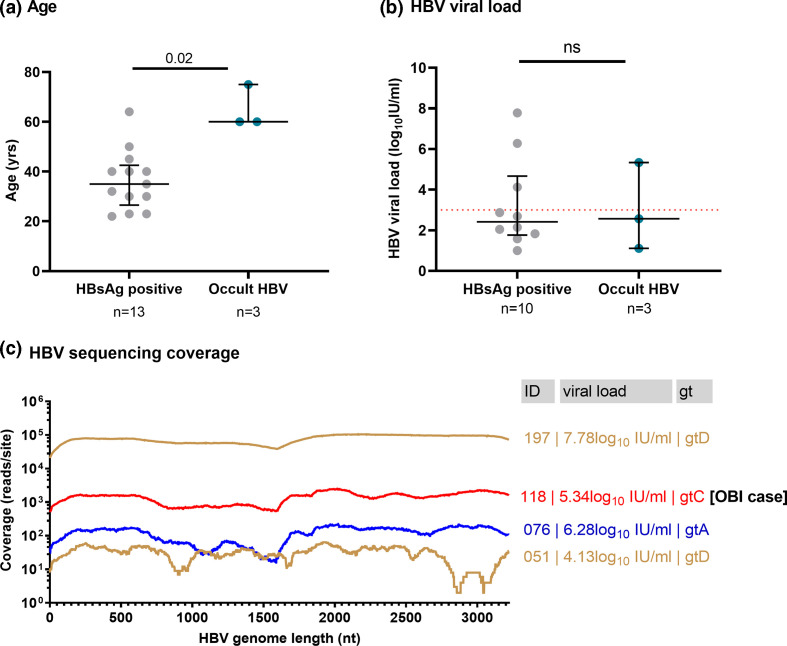
Characteristics of adults in Bangladesh with HBV infection, comparing those with HBsAg-positive infection to those with occult HBV infection and HBV sequencing coverage across the genome. (**a**) Ages and (**b**) viral load of patients with HBsAg-positive HBV and occult HBV infections. Median and interquartile range (IQR) are indicated. The sensitivity threshold for deep sequencing (3.0 log_10_ IU ml^−1^) is indicated in figure b with a dashed red line. Only one of the OBI cases had sufficient viral load for whole genome sequencing (ID 118). (**c**) Plot showing full length coverage of the HBV genome based on sequencing by Illumina. Sample IDs, viral loads and viral genotype (gt) are given for each sequence, with the OBI case highlighted. Median coverage across the genome for the samples ranged from 33 to 77 644 reads per site. Low viral load and small sample volumes were associated with reduced coverage.

### Characteristics of HBV infections and OBI

Median VL of the samples with detectable HBV DNA was 2.6 log_10_ IU ml^−1^ (IQR 1.9–5.0 log_10_ IU ml^−1^), excluding one sample with a VL below the limit of quantification (<1.0 IU ml^−1^). HBeAg testing was available for 12/13 HBV DNA positive samples, of which 1/12 samples (8.3 %; ID 118) was HBeAg-positive. Interestingly this HBeAg-positive sample was also an occult infection. HBV exposure was more common amongst homemakers (*P*=0.006) and less frequent in students (*P*=0.02), but there was no difference according to age or sex (Table S1). OBI arose only in males ≥60 years of age, which is significantly older than the age of the other HBV-positive subjects (Fig. 1a, *P*=0.02). Median HBV VL of the OBI cases (2.6 log_10_ IU ml^−1^, IQR 1.1–5.3 log_10_ IU ml^−1^) was comparable to VL in HBsAg-positive individuals (2.4 log_10_ IU ml^−1^, IQR 1.8–4.7 log_10_ IU ml^−1^) (Fig. 1b, *P*=0.94). One of the OBI infections (sample ID 118) presented with VL 5.3 log_10_ IU ml^−1^, which indicates ongoing high levels of viral replication, despite the absence of HBsAg detection.

### Identification of HBV genotypes A, C and D

**Fig. 2.**
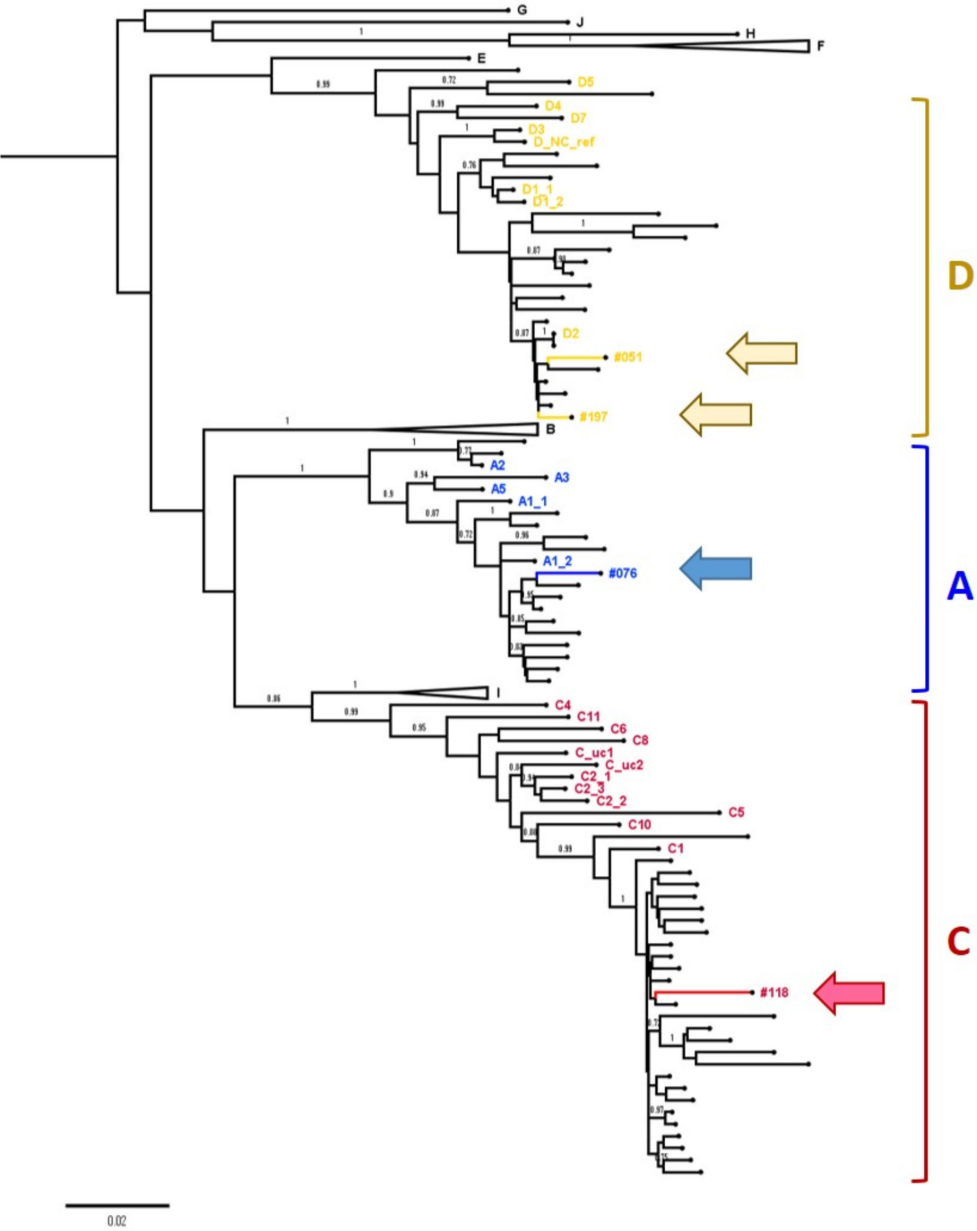
Phylogenetic tree to show the relationship between new HBV sequences and published HBV sequences from Bangladesh. Four full-length HBV consensus sequences generated in this study were analysed alongside HBV genotype reference sequences (for genotypes A-J)^34^ and 61 sequences originating from Bangladesh identified in online databases (unlabelled branches, Table S2). Genotype A sequences are highlighted in blue, genotype C in red and genotype D in yellow. The four sequences generated in this study are indicated with arrows; we identified one genotype C1 sequence (occult infection case, sample 118), one genotype A1 (sample 076) and two genotype D2 sequences (051 and 197). Genotype clades not containing Bangladesh sequences have been collapsed. Bootstrap replicates were repeated 1000 times, and all branches with support >70 % are indicated.

We obtained full length HBV genome sequences from serum from all four individuals with HBV VL ≥3.0 log_10_ IU ml^−1^, which included 1/3 of the OBI cases (ID 118) (Fig. 1c); the remaining two OBI cases had insufficient VL for WGS at 1.1 log_10_ IU ml^−1^ (ID 018) and 2.57 log_10_ IU ml^−1^ (ID 034). Phylogenetic analysis indicated two genotype D infections, and one each of genotypes A and C (Fig. 2). The OBI sequence grouped with other genotype C sequences from Bangladesh (Table S2). Based on the phylogeny of full-length sequences and bootscan analysis, we did not find evidence of dual infection or recombination in this dataset.

The consensus sequence for sample 197 indicated a truncated HBV e-antigen (HBeAg) protein, based on a G1986A mutation present in 57 % reads, but there was insufficient sample to test this sample for HBeAg. The OBI sequence (ID 118) and another sequence (ID 051) also expressed the truncated HBeAg protein as a minority variant, present in 32 % of reads in both samples. However, HBeAg was still detected in sample 118 at the time of testing. This HBeAg truncation is well-described, associated with progression to HBeAg-negativity [37].

### Investigation of OBI sequence polymorphisms

**Fig. 3.**
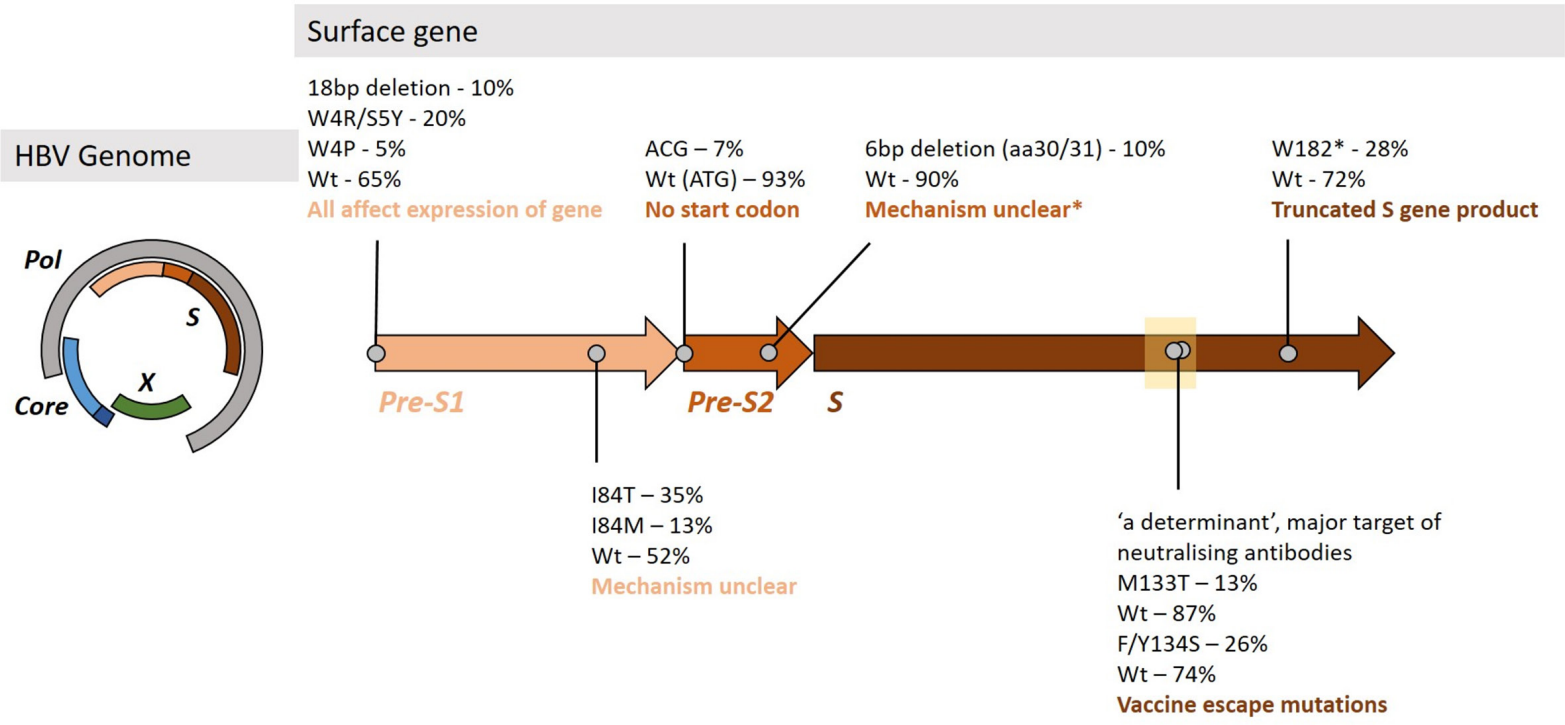
Mutations identified in the Surface gene of sample 118 that may be linked with the OBI phenotype. The HBV surface gene is subdivided into three domains, pre-S1, pre-S2 and S. The proportion of reads containing polymorphisms are shown at various positions throughout the gene. Wt - wild-type, aa - amino acid. Deletions at the start of pre-S1 (causing truncated l-HBs proteins) and highlighted mutations in pre-S1 have all been associated with OBI in previous studies [38, 51, 52]. The mutated start codon of pre-S2 has been linked with an inability to express M-HBs [53]. The ‘a’ determinant (marked with a box at residues 124–147), is a major target of neutralising antibodies and widely-used target for diagnostic assays, and has a strong association with OBI mutations [10, 14, 51, 54]. W182* has been linked to truncated HBsAg products in OBI cases [38], and the mutation is mirrored in the reverse transcriptase gene as V191I (potentially linked to TDF resistance). *The short deletion observed in pre-S2 has not been reported previously and the resulting phenotype is unclear.

We examined the surface gene of OBI sample (ID 118) for the presence of polymorphisms previously linked to OBI [38]. There were no HBsAg (pre-S1, pre-S2 and S) OBI-associated variants in the consensus sequence. However, examining the deep sequencing reads, we identified several potentially relevant sequence changes including pre-S1 and pre-S2 deletions, evidence of truncated S gene products and possible vaccine escape mutations in the ‘a determinant’ [38] (Fig. 3). Analysis indicating the most common haplotypes identified at each site of interest is shown in Fig. S2.

### HBV RAMs VEMs

We examined consensus reverse transcriptase (RT) sequences for RAMs and VEMs (Table 1). RT polymorphisms A181T and Q215P in sample 197 have been associated with 3TC resistance [39]. The overlapping reading frames in HBV mean that the A181T mutation also results in HBsAg W172*, which has been associated with progressive liver disease [40].

**Table 1.** Presence of putative Resistance Associated Mutations (RAMs) and Vaccine Escape Mutations (VEMs) in samples from four adults with chronic HBV infection (including one occult infection, OBI, ID 118), based on analysis of consensus level data and deep sequences generated by Illumina. Positions for codons associated with RAMs are listed in RT, and for VEMs are in HBsAg (sites all numbered using genotype A sequence X02763 as a numbering reference)

Sample ID	HBV genotype	RAMs (consensus sequence)	RAMs (frequency in deep sequence reads)	VEMs (consensus sequence)	VEMs (frequency in deep sequence reads)
051*	D	Not present	Not present	A128V	A128V (> 95 %)
**076**	A	Not present	I233L† (15 %)	Not present	Not present
**118**	C (OBI)	L217R†	V191I† (28 %) L217R† (55%)	Not present	M133T (13 %) F/Y134S (2 6%)
**197**	**D**	Not present	A181T‡ (40 %) Q215P‡ (32 %)	A128V	A128V (> 95 %)

*Sequencing coverage in sample 051 was low (Fig. 1c), so analysis of deep sequence data in this case lacks sensitivity for detection of minor variants.

†Polymorphisms associated with TDF resistance.

‡Polymorphisms associated with 3TC resistance.

Emerging reports suggest that RT polymorphisms can be associated with reduced TDF susceptibility [41]. Among these, L217R was present as consensus in sample 118 (55 % of reads) and V191I was identified as a minority variant (28 % reads) (Table 1). I233L was present as a minority variant (15 % of reads) in sample 076. The clinical significance of these substitutions is not well understood, but phenotypic resistance has most robustly been described in the presence of multiple RT RAMs [32, 33].

VEMs M133T and F/Y134S were present as minority variants in the OBI sequence. A128V, a putative VEM, was identified at consensus level in both our genotype D sequences. However, based on subgenotype (D2 for both 051 and 197), valine is consensus at this position.

## Discussion

### Epidemiology of HBV and OBI infection

Our study identified HBV infection in Bangladeshi adults, with a prevalence of 8 % in a tertiary hospital cohort, of which 3/16 (18.8 %) were OBI. Existing sequence data from Bangladesh suggest genotypes C and D each account for ~40 % of the total burden, and genotype A for the remainder, with a high proportion of recombinants and mixed genotype infections [3, 5, 42]. Of note, we identified a genotype A1 isolate, more typically associated with transmission in Southern and Eastern Africa, although potentially increasingly prevalent in Asia [3, 5].

The lower exposure of students may reflect younger age and better socioeconomic backgrounds, while OBI was more common in older men. However, we cannot make generalisations about HBV distribution in this setting, based on a small cohort recruited in a very specific clinical context. Larger studies recruiting a more generalisable population are required, along with the incorporation of anti-HBs screening to determine the impact of vaccination.

### Defining and characterising OBI

Definitions of OBI vary, and identifying cases is dependent on the sensitivity of the platform in use for HBV DNA detection. Sequencing data from a case of OBI demonstrated a combination of mutations and deletions, occurring in all three regions of the surface gene. None of the deletions was present at consensus level, illustrating the importance of deep sequencing data for identifying minority variants that may be driving uncommon phenotypes. Several of the mutations identified have been linked with the progression of severe liver disease, including W182*, thought to interfere with cell cycle regulation [43], typically in the context of low VL [40].

Short read sequencing provides advantages in sequencing depth, and deletion detection but the reconstruction of full-length haplotypes remains challenging, making it difficult to determine linkage between mutations. This question could potentially be addressed with longer reads that can be obtained through PCR and Sanger sequencing, but unfortunately, we were constrained by limited sample volumes. Given that all mutations associated with OBI were identified only as minority variants; to produce an OBI phenotype this suggests that all genomes must carry at least one of the relevant mutations, but understanding of how these mutations interact to produce OBI is limited. Developing sensitive long-read sequencing approaches for HBV remains an important aspiration, enabling improved understanding of the interactions between different polymorphisms, and their contribution to disease states [44].

### Evidence for drug and vaccine resistance

A previous meta-analysis of drug resistance in Bangladesh reported a prevalence of 3TC resistance of 11 %, but no TDF resistant motifs [35]. Although our cohort is small, it is striking that we nevertheless identified mutations linked to both 3TC and TDF resistance. In these treatment-naive patients we cannot confirm the *in vivo* significance of these RAMs, but our data do raise concern that these mutations are circulating; further work is needed to explore their prevalence and clinical significance.

Mutations in HBsAg at positions 133 and 134 are localised within a region known to contain major B-cell epitopes, and can therefore contribute to vaccine resistance [13]. A128V is a potential VEM, although its specific contribution to vaccine escape remains uncertain [45]. Previous reports of VEMs in Bangladesh [35], and a case report of a child [36], have also identified the A128V substitution, and we identified this variant in two of our sequences (both subgenotype D2). However, a subgenotype D2 reference sequence (MF925358) [30] has 128-valine, suggesting it is the most common residue in this subgenotype. This raises the possibility that subgenotype D2 might be more susceptible to vaccine resistance, but further work is required to substantiate this. The significance of these variants in our sequence data is uncertain, as we did not collect vaccine history data or measure vaccine-mediated antibody titres.

### Caveats and limitations

We have undertaken HBV screening on only a small cohort and without longitudinal follow-up. Although HBV was presumed to be incidental to the reasons for presentation to hospital, we cannot exclude a selection bias. HBV infections were likely to be chronic, but we did not distinguish between anti-HBc IgM and IgG, so acute infection is also possible. Routine liver function tests were not available (and interpretation would have been confounded by acute illness). Subjects were not routinely screened for HCV, which has been associated with occult HBV infection and has an estimated prevalence of 1 % in this region [6, 46].

The study highlights the current technical challenges of generating whole genome deep sequencing data for HBV, for which a typical threshold VL is >3.0 log_10_ IU ml^−1^ [47, 48]. The median VL of 2.6 log_10_ IU ml^−1^ in this cohort illustrates the high proportion of cases with VL below the current sequencing sensitivity [48, 49]. For this reason, we generated full length sequence data in only 4/13 cases, leaving the majority of HBV sequences ‘under the radar’, including two OBI. Previous studies have indicated there can be multiple pathways driving occult phenotypes, including low HBsAg production (often in the context of correspondingly low VL), anti-HBs seroconversion, and ‘false-occult’ infections, associated with the failure of diagnostic tests [8]. Whilst the high VL of the OBI we sequenced may be suggestive of a diagnostic failure, the lack of highly sensitive sequencing methods for HBV limits our insight into the mechanisms behind the other two OBI cases. HBsAg testing is potentially more sensitive for HBV detection as HBsAg can be detectable in the absence of HBV DNA. However, sensitivities vary between assay platforms, and both biomarkers are imperfect proxies for inferring disease process in the liver. The use of an alternative HBsAg detection assay (potentially lowering the HBsAg LoD down to 0.005 IU ml^−1^) may have been informative [8]. These technical challenges, along with a paucity of well-studied longitudinal cohorts, remain barriers in furthering our understanding of HBV disease and transmission.

### Implications for policy and clinical practice

The approach we adopted demonstrates the feasibility of routine HBV screening for adults admitted to hospital in Bangladesh. Improvements in ascertainment are key to advancing progress towards international elimination goals [6], while for individual patients facilitates referral into appropriate clinical care. Our data also highlight the importance of continued deployment of vaccination programmes with a focus on infant coverage [50]. Given the high burden of HBV infection in Bangladesh, there is an urgent need to expand our understanding of population and molecular epidemiology, in order to strengthen advocacy, education and research, and to inform investment in clinical care and public health.

### Data availability

The datasets generated during and/or analysed during the current study are available in the following repositories:

- GenBank accession numbers MT114170 - MT114173.
- Metadata file of demographic data and HBV screening results in the fever cohort (*n*=201 subjects) on Figshare - DOI: https://doi.org/10.6084/m9.figshare.11973930.
- A STROBE statement has been submitted as a supplementary file with this manuscript.

## Supplementary Data

Supplementary material 1

## References

[1] WHO (2017). Global hepatitis report, 2017 [Internet]. World Health Organization; [cited 2019 Apr 24]. http://www.who.int/hepatitis/publications/global-hepatitis-report2017/en.

[2] WHO (2017). Global health sector strategy on viral hepatitis 2016-2021 [Internet]. World Health Organization. http://www.who.int/hepatitis/strategy2016-2021/ghss-hep/en.

[3] Munshi SU, Tran TTT, TNT V, Tabassum S, Sultana N (2017). Molecular characterization of hepatitis B virus in Bangladesh reveals a highly recombinant population. PLoS One.

[4] Al Mahtab M (2017). Elimination of hepatitis viruses: Bangladesh scenario. Euroasian J Hepatogastroenterol.

[5] Uz-Zaman MH, Rahman A, Yasmin M (2018). Epidemiology of hepatitis B virus infection in Bangladesh: Prevalence among general population, risk groups and genotype distribution. Genes (Basel).

[6] Cooke GS, Andrieux-Meyer I, Applegate TL, Atun R, Burry JR (2019). Accelerating the elimination of viral hepatitis: a Lancet Gastroenterology & Hepatology commission. Lancet Gastroenterol Hepatol.

[7] Childs L, Roesel S, Tohme RA (2018). Status and progress of hepatitis B control through vaccination in the South-East Asia Region, 1992-2015. Vaccine.

[8] Raimondo G, Locarnini S, Pollicino T, Levrero M, Zoulim F (2019). Update of the statements on biology and clinical impact of occult hepatitis B virus infection. J Hepatol.

[9] Martin CM, Welge JA, Rouster SD, Shata MT, Sherman KE (2012). Mutations associated with occult hepatitis B virus infection result in decreased surface antigen expression in vitro. J Viral Hepat.

[10] Kim MH, Kang SY, Lee WI (2017). Occult HBV among Anti-hbc alone: Mutation analysis of an HBV surface gene and pre-S gene. Yonsei Med J.

[11] Malagnino V, Fofana DB, Lacombe K, Gozlan J (2018). Occult Hepatitis B virus infection: An old entity with novel clinical involvements. Open Forum Infect Dis.

[12] Huo TI, Wu JC, Lee PC, Chau GY, Lui WY (1998). Sero-clearance of hepatitis B surface antigen in chronic carriers does not necessarily imply a good prognosis. Hepatology.

[13] Colagrossi L, Hermans LE, Salpini R, Di Carlo D, Pas SD (2018). Immune-escape mutations and stop-codons in HBsAg develop in a large proportion of patients with chronic HBV infection exposed to anti-HBV drugs in Europe. BMC Infect Dis.

[14] Coleman PF (2006). Detecting hepatitis B surface antigen mutants. Emerg Infect Dis.

[15] Mahtab MA, Akbar SMF, Rahman S (2012). Hepatitis B surface antigen-negative, but HBV DNA-positive patients in Bangladesh. Bangladesh Med Res Counc Bull.

[16] Jahan M, Islam MA, Akbar SMF, Takahashi K, Tabassum S (2016). Anti-hbc screening of blood donors in Bangladesh: Relevance to containment of HBV propagation. J Clin Exp Hepatol.

[17] L-P H, Liu DP, Chen QY, Harrison TJ, He X (2015). Occult HBV infection may be transmitted through close contact and manifest as an overt infection. PLOS ONE.

[18] Candotti D, Lin CK, Belkhiri D, Sakuldamrongpanich T, Biswas S (2012). Occult hepatitis B infection in blood donors from South East Asia: molecular characterisation and potential mechanisms of occurrence. Gut.

[19] Center for Biologics Evaluation, Research (2019). Complete list of DSA for infectious agents and HIV diagnostic assays [Internet]. U.S. Food and Drug administration. https://www.fda.gov/vaccines-blood-biologics/complete-list-donor-screening-assays-infectious-agents-and-hiv-diagnostic-assays.

[20] Allice T, Cerutti F, Pittaluga F, Varetto S, Gabella S (2007). COBAS AmpliPrep-COBAS TaqMan hepatitis B virus (HBV) test: a novel automated real-time PCR assay for quantification of HBV DNA in plasma. J Clin Microbiol.

[21] Thomson E, Ip CLC, Badhan A, Christiansen MT, Adamson W (2016). Comparison of next-generation sequencing technologies for comprehensive assessment of full-length hepatitis C viral genomes. J Clin Microbiol.

[22] Podlaha O, Gane E, Brunetto M, Fung S, Chuang WL (2019). Large-scale viral genome analysis identifies novel clinical associations between Hepatitis B virus and chronically infected patients. Sci Rep.

[23] Martel N, Gomes SA, Chemin I, Trépo C, Kay A (2013). Improved rolling circle amplification (RCA) of hepatitis B virus (HBV) relaxed-circular serum DNA (RC-DNA. J Virol Methods.

[24] Martin M (2011). Cutadapt removes adapter sequences from high-throughput sequencing reads. EMBnet J.

[25] Langmead B, Salzberg SL (2012). Fast gapped-read alignment with Bowtie 2. Nat Methods.

[26] Li H, Durbin R (2009). Fast and accurate short read alignment with Burrows-Wheeler transform. Bioinformatics.

[27] Simmonds P (2012). SSE: a nucleotide and amino acid sequence analysis platform. BMC Res Notes.

[28] Kumar S, Stecher G, Tamura K (2016). MEGA7: Molecular Evolutionary Genetics Analysis Version 7.0 for Bigger Datasets. Mol Biol Evol.

[29] (2020). FigTree [Internet]. [cited 2020 Jan 23]. Available from. http://tree.bio.ed.ac.uk/software/figtree.

[30] McNaughton AL, Revill PA, Littlejohn M, Matthews PC, Ansari MA (2020). Analysis of genomic-length HBV sequences to determine genotype and subgenotype reference sequences. J Gen Virol.

[31] Martin DP, Murrell B, Golden M, Khoosal A, Muhire B (2015). RDP4: Detection and analysis of recombination patterns in virus genomes. Virus Evol.

[32] Beloukas A, Geretti AM (2017). Hepatitis B Virus Drug Resistance. Antimicrobial Drug Resistance.

[33] Mokaya J, McNaughton AL, Bester PA, Goedhals D, Barnes E (2020). Hepatitis B virus resistance to tenofovir: fact or fiction? A synthesis of the evidence to date. Wellcome Open Res.

[34] Raheel M, Choga WT, Blackard JT (2020). The distribution of hepatitis B virus surface antigen polymorphisms at positions associated with vaccine escape. J Med Virol.

[35] Hossain MG, Ueda K (2019). A meta-analysis on genetic variability of RT/HBsAg overlapping region of hepatitis B virus (HBV) isolates of Bangladesh. Infect Agent Cancer.

[36] Shaha M, Hadisur Rahman M, Jahan M, Dey SK, Das KC (2018). Identification of a novel tri-genotypic recombinant Hepatitis B virus in Bangladesh. Virus Res.

[37] Manesis EK (2006). HBeAg-negative chronic hepatitis B: from obscurity to prominence. J Hepatol.

[38] Kim H, Lee SA, Kim DW, Lee SH, Kim BJ (2013). Naturally occurring mutations in large surface genes related to occult infection of hepatitis B virus genotype c. PLOS ONE.

[39] Mokaya J, McNaughton AL, Hadley MJ, Beloukas A, Geretti AM (2018). A systematic review of hepatitis B virus (HBV) drug and vaccine escape mutations in Africa: A call for urgent action. PLoS Negl Trop Dis.

[40] Lee SA, Kim H, Kim H, Kim BJ (2012). Nucleotide change of codon 182 in the surface gene of hepatitis B virus genotype C leading to truncated surface protein is associated with progression of liver diseases. J Hepatol.

[41] Mokaya J, Maponga TG, McNaughton AL, Van Schalkwyk M, Hugo S (2020). Evidence of tenofovir resistance in chronic hepatitis B virus (HBV) infection: An observational case series of South African adults. J Clin Virol.

[42] Rahman MA, Hakim F, Ahmed M, Ahsan CR, Nessa J (2016). Prevalence of genotypes and subtypes of hepatitis B viruses in Bangladeshi population. Springerplus.

[43] Wang ML, Wu D-B, Tao YC, Chen LL, Liu CP (2018). The truncated mutant HBsAg expression increases the tumorigenesis of hepatitis B virus by regulating TGF-β/Smad signaling pathway. Virol J.

[44] McNaughton AL, Roberts HE, Bonsall D, de Cesare M, Mokaya J (2019). Illumina and Nanopore methods for whole genome sequencing of hepatitis B virus (HBV. Sci Rep.

[45] Yan B, Lv J, Feng Y, Liu J, Ji F (2017). Temporal trend of hepatitis B surface mutations in the post-immunization period: 9 years of surveillance (2005-2013) in eastern China. Sci Rep.

[46] Mamun-Al M (2016). Past, present, and future of viral hepatitis in Bangladesh. Euroasian J Hepatogastroenterol.

[47] Astbury S, Da Costa Nunes Soares MM, Peprah E, King BJ, Jardim ACG (2019). Extraction-free direct PCR from dried serum spots permits HBV genotyping and RAS identification by Sanger and minION sequencing. biorxiv.

[48] McNaughton AL, D’Arienzo V, Ansari MA, Lumley SF, Littlejohn M (2019). Insights from deep sequencing of the HBV genome–unique, tiny, and misunderstood. Gastroenterology.

[49] Downs LO, Matthews P, Vawda S, Bester A, Lythgoe K (2020). Bimodal distribution and set point HBV DNA viral loads in chronic infection: retrospective analysis of cohorts from the UK and South Africa. Wellcome Open Res.

[50] Tamandjou CR, Maponga TG, Chotun N, Preiser W, Andersson MI (2017). Is hepatitis B birth dose vaccine needed in Africa. Pan Afr Med J.

[51] Kim H, Kim B-J (2015). Association of preS/S mutations with cccult Hepatitis B Virus (HBV) infection in South Korea: transmission potential of distinct cccult HBV variants. Int J Mol Sci.

[52] Wang J, Zhang P, Zeng J, Du P, Zheng X (2020). Occurrence of occult hepatitis B virus infection associated with envelope protein mutations according to anti-HBs carriage in blood donors. IntJ Infect Dis.

[53] Pollicino T, Raffa G, Costantino L, Lisa A, Campello C (2007). Molecular and functional analysis of occult hepatitis B virus isolates from patients with hepatocellular carcinoma. Hepatology.

[54] Javanmard D, Namaei MH, Farahmand M, Ziaee A, Amini E (2019). Molecular and serological characterization of occult hepatitis B virus infection among patients with hemophilia. J Med Virol.

