# Supplementary material for: Endemic HBV among hospital in-patients in Bangladesh, including evidence of occult infection
Source: J Gen Virol. 2021 Jul 30;102(7):001628. doi: 10.1099/jgv.0.001628 (PMC8491891; doi:10.1099/jgv.0.001628)
Supplement: Supplementary material 1 [file jgv-102-1628-s001.pdf]

**SUPPLEMENTARY MATERIAL**

# Endemic HBV among hospital in-patients in Bangladesh, including evidence of occult infection

Fazle Rabbi Chowdhury, Anna L McNaughton, Mohammad Robed Amin,
Lovely Barai, Mili Rani Saha, Tanjila Rahman, Bikash Chandra Das,
Md Rokibul Hasan, K.M. Shahidul Islam, M A Faiz, Mamun Al-Mahtab,
Jolynne Mokaya, Katie Jeffery, Monique Andersson, Mariateresa de Cesare,
M Azim Ansari, Susanna Dunachie, Philippa C Matthews

**Suppl Table 1: Characteristics of patients recruited into a fever cohort at two teaching hospital centres in Dhaka, Bangladesh.**

| | HBV positive (HBsAg or HBV DNA positive) | Resolved HBV (anti-HBc positive only) | HBV negative (anti-HBc negative) | p-value[a] |
|---|---|---|---|---|
| **Number of patients** *(%)* | 16 (7.9%) | 56 (27.9%) | 129 (64.2%) | - |
| **Median age in yrs** *(IQR)* | 40 (30-58) | 38 (28-60) | 34 (21-55) | 0.15 |
| **Male sex,** *n (%)* | 10 (62.5%) | 33 (58.9%) | 72 (55.8%) | 0.35 |
| **Median distance in km travelled to hospital** *(IQR)* | 135 (28-150) | 70 (16-200) | 100 (28-150) | 0.81 |
| **Median number of days between symptom onset and hospitalization** *(IQR)* | 10 (7-12) | 10 (6-15.8) | 10 (6-15) | 0.18 |
| **Occupation** *n (%)*[b] | | | | |
| **Homemaker** | 4 (25.0%) | 20 (35.7%) | 19 (14.7%) | 0.006 |
| **Student** | 1 (6.3%) | 10 (17.9%) | 41 (31.7%) | 0.02 |
| **Farmer / day labourer** | 8 (50%) | 22 (39.3%) | 43 (33.3%) | 0.28 |
| **Others**[c] | 3 (18.7%) | 4 (7.1%) | 26 (20.1%) | 0.09 |
| **Outcome** *n (%)* | | | | |
| **Died in hospital**[d] | 3 (18.8%) | 8 (14.3%) | 34 (26.3%) | 0.18 |

[a] One-way Anova and Fisher's exact test (two tailed) was applied

[b] p-values not corrected for multiple comparisons; based on a Bonferroni approach, only being a homemaker would remain significantly associated with HBV status.

[c] Includes businessman, carpenter, plumber and unemployed

[d] All other patients were either discharged (n=154) or they absconded during treatment (n=2) in hospital

**Suppl Table 2: Accession numbers of full-length HBV sequences identified in GenBank originating from Bangladesh.** The sequences were analysed alongside the four consensus sequences generated from our study (Fig 2).

| **Accession numbers** |
|---|
| MK628732.1, MH220970.1, MH220971.1, MG725248.1, MF925358.1, MF925359.1, MF925360.1, MF925361.1, MF925362.1, MF925363.1, MF925364.1, MF925365.1, MF925366.1, MF925367.1, MF925368.1, MF925369.1, MF925370.1, MF925371.1, MF925372.1, MF925373.1, MF925374.1, MF925375.1, MF925376.1, MF925377.1, MF925378.1, MF925379.1, MF925380.1, MF925381.1, MF925382.1, MF925383.1, MF925384.1, MF925385.1, MF925386.1, MF925387.1, MF925388.1, MF925389.1, MF925390.1, MF925391.1, MF925392.1, MF925393.1, MF925394.1, MF925395.1, MF925396.1, MF925397.1, MF925398.1, MF925399.1, MF925400.1, MF925401.1, MF925402.1, MF925403.1, MF925404.1, MF925405.1, MF925406.1, MF925407.1, MF925408.1, MF925409.1, MF925410.1, AB116082.1, AB116083.1, AB116084.1, AB116085.1 |

**Suppl Figure 1: Flow chart illustrating the results of screening for HBV markers in serum from adults in a Bangladesh fever cohort.** All patients were screened for both anti-HBc and HBsAg. All HBsAg positive patients were further screened for HBV DNA. A total of 16 patients were HBV positive, with three different serological profiles; (a) HBsAg positive, HBV DNA positive; (b) HBsAg positive, HBV DNA negative; (c) HBsAg negative, HBV DNA positive (occult HBV infection). *One patient was anti-HBc negative, HBsAg positive and HBV DNA negative. This patient was likely a false reactive on the HBsAg screening test and considered to be HBV negative for the study, although there is a possibility that this was an early acute stage infection.

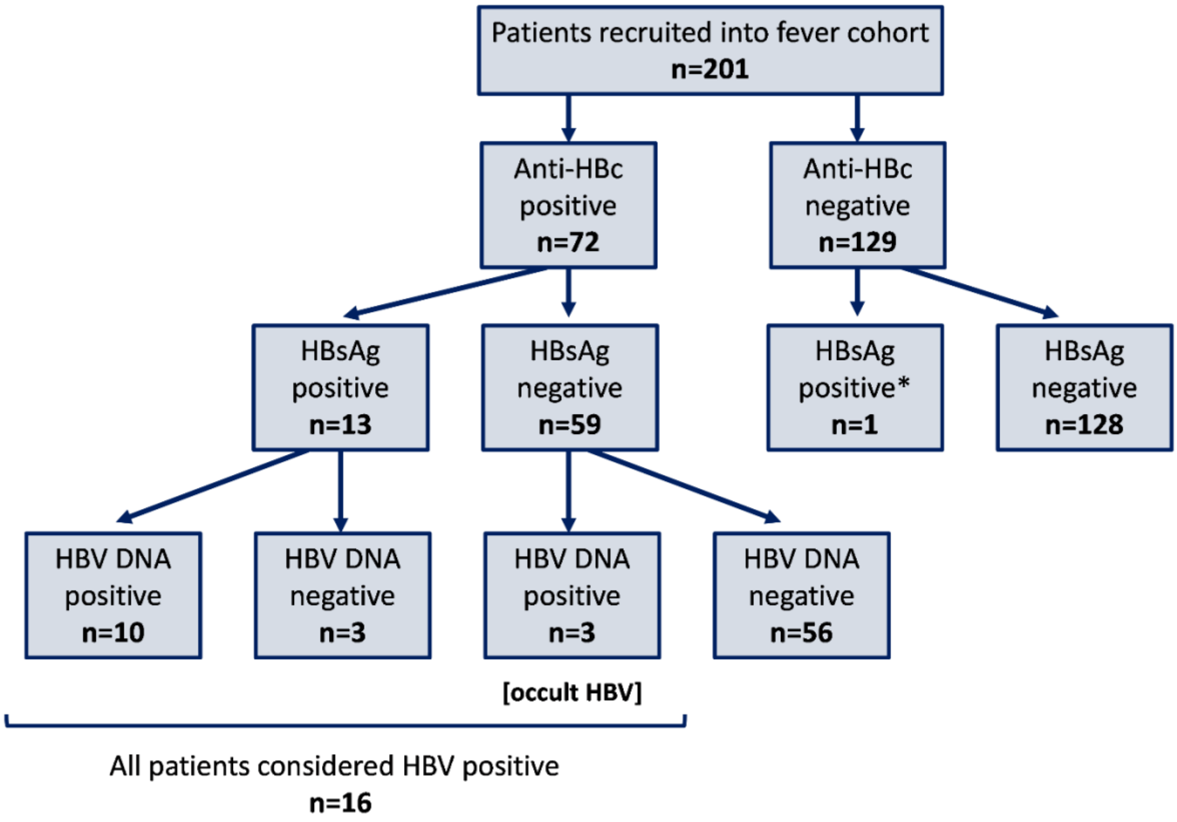

**Suppl Figure 2: Nucleotide haplotypes and amino acid translations for OBI-associated polymorphisms in sample ID 118.** The most common nucleotide sequences identified in the deep sequencing reads at sites highlighted in Figure 3 are shown. Mutations of interest are indicated with red arrows. The number of reads identified with the sequence is indicated along with the proportion of reads (%). Only haplotypes occurring at a frequency of ≥5% (or could be rounded up to 5%) are shown. Proportions do not total to 100% as large numbers of reads had variants occurring at <5%. Frequencies may also differ from those shown in Figure 3 as the polymorphisms highlighted in Figure 3 can occur in multiple haplotype sequences, many of which are not shown in this figure.

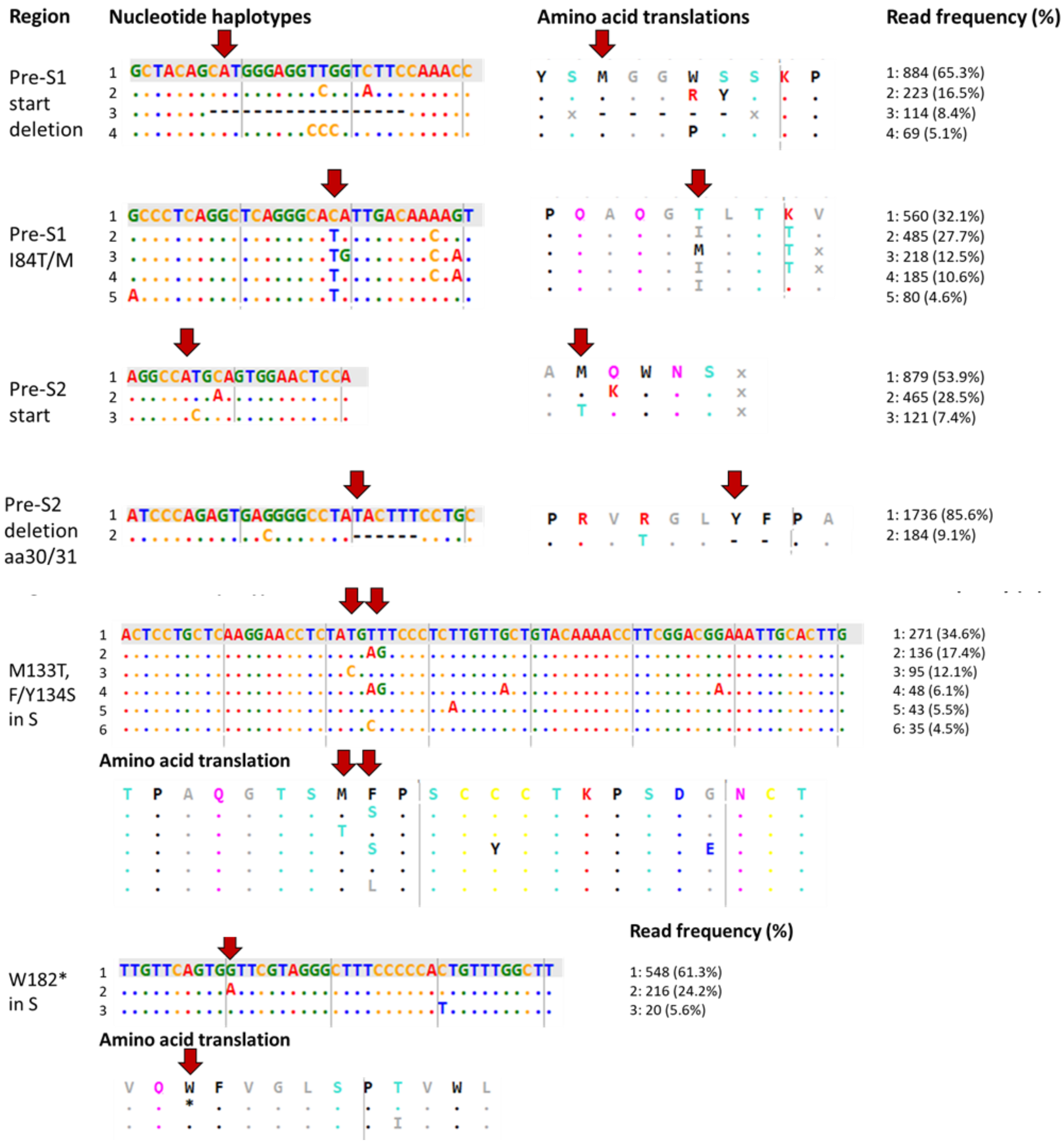

STROBE Statement—Checklist of items that should be included in reports of ***cross-sectional studies***

| | Item No | Recommendation | Manuscript section |
|---|---|---|---|
| **Title and abstract** | 1 | (*a*) Indicate the study's design with a commonly used term in the title or the abstract | Title |
| | | (*b*) Provide in the abstract an informative and balanced summary of what was done and what was found | Abstract |
| **Introduction** | | | |
| Background/rationale | 2 | Explain the scientific background and rationale for the investigation being reported | Introduction |
| Objectives | 3 | State specific objectives, including any pre-specified hypotheses | Introduction, final paragraph |
| **Methods** | | | |
| Study design | 4 | Present key elements of study design early in the paper | Methods |
| Setting | 5 | Describe the setting, locations, and relevant dates, including periods of recruitment, exposure, follow-up, and data collection | Methods, subsection 'Study settings and clinical cohort' |
| Participants | 6 | (*a*) Give the eligibility criteria, and the sources and methods of selection of participants | Methods, subsection 'Study settings and clinical cohort' |
| Variables | 7 | Clearly define all outcomes, exposures, predictors, potential confounders, and effect modifiers. Give diagnostic criteria, if applicable | Methods, subsection 'Blood sampling and baseline screening for HBV infection' |
| Data sources/ measurement | 8* | For each variable of interest, give sources of data and details of methods of assessment (measurement). Describe comparability of assessment methods if there is more than one group | Methods, subsections 'DNA extraction and sequencing', 'Analysis of sequence data', 'Identification of potential resistance associated mutations (RAMs) and vaccine escape mutations (VEMs)' |
| Bias | 9 | Describe any efforts to address potential sources of bias | Methods and Discussion subsection 'Caveats and limitations' |
| Study size | 10 | Explain how the study size was arrived at | Methods, subsection 'Study settings and clinical cohort' |
| Quantitative variables | 11 | Explain how quantitative variables were handled in the analyses. If applicable, describe which groupings were chosen and why | Methods, subsection, 'Statistics' |
| Statistical methods | 12 | (*a*) Describe all statistical methods, including those used to control for confounding | Methods, subsections 'Statistics' |
| | | (*b*) Describe any methods used to examine subgroups and interactions | Methods, subsections 'Identification of potential resistance associated mutations (RAMs) and vaccine escape mutations (VEMs)', 'Statistics' |

| | | | |
|---|---|---|---|
| | | (*c*) Explain how missing data were addressed | Methods |
| | | (*d*) If applicable, describe analytical methods taking account of sampling strategy | Methods |
| | | (*e*) Describe any sensitivity analyses | n/a |
| **Results** | | | |
| Participants | 13* | (a) Report numbers of individuals at each stage of study—eg numbers potentially eligible, examined for eligibility, confirmed eligible, included in the study, completing follow-up, and analysed | Fig 1, Table 1, Methods, Metadata on Figshare (DOI: 10.6084/m9.figshare.11973930) |
| | | (b) Give reasons for non-participation at each stage | n/a |
| | | (c) Consider use of a flow diagram | Fig 1 |
| Descriptive data | 14* | (a) Give characteristics of study participants (eg demographic, clinical, social) and information on exposures and potential confounders | Table 1, Metadata on Figshare (DOI: 10.6084/m9.figshare.11973930) |
| | | (b) Indicate number of participants with missing data for each variable of interest | Fig 1, Table 1, Metadata on Figshare (DOI: 10.6084/m9.figshare.11973930) |
| Outcome data | 15* | Report numbers of outcome events or summary measures | Table 1, Results subsections 'Cohort description and HBV epidemiology', 'Characteristics of HBV infection' |
| Main results | 16 | (*a*) Give unadjusted estimates and, if applicable, confounder-adjusted estimates and their precision (eg, 95% confidence interval). Make clear which confounders were adjusted for and why they were included | n/a |
| | | (*b*) Report category boundaries when continuous variables were categorized | n/a |
| | | (*c*) If relevant, consider translating estimates of relative risk into absolute risk for a meaningful time period | n/a |
| Other analyses | 17 | Report other analyses done—eg analyses of subgroups and interactions, and sensitivity analyses | Table 2, Results subsections 'Identification of HBV genotypes A, C and D', 'HBV Resistance Associated Mutations (RAMs)', 'Investigation of sequence data from patient with OBI' |
| **Discussion** | | | |
| Key results | 18 | Summarise key results with reference to study objectives | Discussion |
| Limitations | 19 | Discuss limitations of the study, taking into account sources of potential bias or imprecision. Discuss both direction and magnitude of any potential bias | Discussion subsection 'Caveats and limitations' |

| | | | |
|---|---|---|---|
| Interpretation | 20 | Give a cautious overall interpretation of results considering objectives, limitations, multiplicity of analyses, results from similar studies, and other relevant evidence | Discussion |
| Generalisability | 21 | Discuss the generalisability (external validity) of the study results | Discussion |
| **Other information** | | | |
| Funding | 22 | Give the source of funding and the role of the funders for the present study and, if applicable, for the original study on which the present article is based | Funding subsection |

*Give information separately for exposed and unexposed groups.

**Note:** An Explanation and Elaboration article discusses each checklist item and gives methodological background and published examples of transparent reporting. The STROBE checklist is best used in conjunction with this article (freely available on the Web sites of PLoS Medicine at http://www.plosmedicine.org/, Annals of Internal Medicine at http://www.annals.org/, and Epidemiology at http://www.epidem.com/). Information on the STROBE Initiative is available at www.strobe-statement.org.
